# Interleukin-1β Triggers p53-Mediated Downmodulation of CCR5 and HIV-1 Entry in Macrophages through MicroRNAs 103 and 107

**DOI:** 10.1128/mBio.02314-20

**Published:** 2020-09-29

**Authors:** Robert Lodge, Nicolas Bellini, Mélanie Laporte, Syim Salahuddin, Jean-Pierre Routy, Petronela Ancuta, Cecilia T. Costiniuk, Mohammad-Ali Jenabian, Éric A. Cohen

**Affiliations:** a Laboratory of Human Retrovirology, Institut de Recherches Cliniques de Montréal, Montreal, Quebec, Canada; b Department of Biological Sciences, Université du Québec à Montréal, Montreal, Quebec, Canada; c Research Institute of the McGill University Health Centre, Montreal, Quebec, Canada; d Division of Hematology, Department of Medicine, McGill University Health Centre, Montreal, Quebec, Canada; e Chronic Viral Illness Service, Department of Medicine, McGill University Health Centre, Montreal, Quebec, Canada; f Centre de Recherche du Centre Hospitalier de l’Université de Montréal, Montreal, Quebec, Canada; g Department of Microbiology, Infectiology and Immunology, Faculty of Medicine, Université de Montréal, Montreal, Quebec, Canada; h Division of Infectious Diseases, Department of Medicine, McGill University Health Centre, Montreal, Quebec, Canada; i Department of Microbiology and Immunology, Faculty of Medicine, McGill University, Montreal, Quebec, Canada; Rutgers-Robert Wood Johnson Medical School

**Keywords:** alveolar macrophage, CCR5, CD4, colon macrophage, HIV reservoir, IL-1β, inflammation, microRNA, virus receptor, p53

## Abstract

Macrophages are a target of human immunodeficiency virus type 1 (HIV-1) and may serve as a viral reservoir during antiretroviral therapy (ART). Their susceptibility to HIV-1 infection is subject to variations from permissiveness to resistance depending on their origin, tissue localization, and polarization profile. This is in part due to the expression of regulatory microRNAs. Here, we identify two microRNA paralogs, microRNA 103 (miR-103) and miR-107, as regulators of CCR5 expression that are upregulated in noninfected bystander cells of HIV-1-infected-monocyte-derived macrophage (MDM) cultures. Transfection of microRNA 103 mimics in MDMs reduced CCR5 expression levels and inhibited CCR5-dependent HIV-1 entry, whereas the corresponding antagomirs enhanced virus spread in HIV-infected MDMs. Treatment of MDMs with interleukin-1β (IL-1β) enhanced microRNA 103 expression, a condition that we found contributed to the reduction of CCR5 mRNA in IL-1β-exposed MDMs. Interestingly, we show that the induction of miR-103/107 expression is part of a tumor suppressor p53 response triggered by secreted IL-1β that renders macrophages refractory to HIV-1 entry. In a more physiological context, the levels of microRNAs 103 and 107 were found enriched in tissue-resident colon macrophages of healthy donors and alveolar macrophages of individuals under antiretroviral therapy, conceivably contributing to their relative resistance to HIV-1 infection. Overall, these findings highlight the role of p53 in enforcing proinflammatory antiviral responses in macrophages, at least in part, through miR-103/107-mediated downmodulation of CCR5 expression and HIV-1 entry.

## Abstract (precis)

Macrophages are heterogeneous immune cells that display varying susceptibilities to HIV-1 infection, in part due to the expression of small noncoding microRNAs involved in the posttranscriptional regulation of gene expression and silencing. Here, we identify microRNAs 103 and 107 as important p53-regulated effectors of the antiviral response triggered by the proinflammatory cytokine IL-1β in macrophages. These microRNAs, which are enriched in colon macrophages of healthy donors and alveolar macrophages of HIV-infected individuals under antiretroviral therapy, act as inhibitors of HIV-1 entry through their capacity to downregulate the CCR5 coreceptor. These results highlight the important role played by miR-103/107 in modulating CCR5 expression and HIV-1 entry in macrophages. They further underscore a distinct function of the tumor suppressor p53 in enforcing proinflammatory antiviral responses in macrophages, thus providing insight into a cellular pathway that could be targeted to limit the establishment of viral reservoirs in these cells.

## INTRODUCTION

In addition to CD4, the primary receptor of human immunodeficiency virus type 1 (HIV-1), chemokine receptors are essential coreceptors for virus entry. Among these chemokine receptors, CCR5 serves as a major coreceptor for HIV-1 (1, 2). Variation in its expression impacts disease outcomes after HIV-1 infection (3), and its reduction at the cell surface, such as that resulting from a 32-bp deletion in the *CCR5* gene, entails resistance to HIV-1 infection (4). As a matter of fact, the degree of CCR5 surface expression influences various facets of HIV pathogenesis, such as transmission and virus burden, but also the efficiency of CCR5 blockers and entry inhibitors in treatments as well as virus neutralization by HIV-1-specific antibodies (Abs) (3).

Macrophages are increasingly recognized as an important cellular target of HIV/simian immunodeficiency virus (SIV) infection at different stages of disease (5–10). Although they express several chemokine receptors, CCR5 is the main coreceptor used by HIV-1 to infect these myeloid cells (11). Given their life span ranging from months to years and their unique ability to resist HIV-1 cytopathic effects and CD8^+^ T cell-mediated killing, macrophages have been proposed to be an important sanctuary for HIV-1 and a potential viral reservoir during antiretroviral therapy (ART) (12–14). While HIV-1 infection has been demonstrated in lymphoid as well as nonlymphoid tissues, tissue-resident macrophages display different susceptibilities to productive HIV-1 infection (14). For instance, whereas productive infection has been observed in vaginal (15), penile urethral (16, 17), and, to a weaker degree, lung alveolar (18–21) macrophages, intestinal macrophages reveal significant resistance to HIV-1 infection (22, 23), thus indicating that the origin and activation status of tissue-resident macrophages as well as local environmental signals influence their susceptibility to HIV-1 infection. Indeed, the nonpermissiveness of intestinal macrophages is in part due to reduced expression levels of CD4 and CCR5 (22). Reduced cell surface CD4 and CCR5 expression levels are also observed in HIV-1-resistant monocyte-derived macrophages (MDMs) that are activated by cytokines such as tumor necrosis factor alpha (TNF-α) or interferon gamma (IFN-γ) (24–27). Nevertheless, other cytokine-induced antiviral factors such as SAMHD1, APOBEC3G, TRIM5α, TRIM22, and BST2/tetherin also contribute to restrict HIV-1 infection in activated macrophages to various degrees (28). A comprehensive understanding of host factors and signals modulating the susceptibility of macrophages to HIV-1 infection is likely to provide important insight into cellular pathways that could be targeted to limit the establishment and persistence of viral reservoirs in macrophages.

MicroRNAs have been shown to play a key role in modulating the susceptibility of HIV target cells to HIV-1 infection (29). The 3′ untranslated region (UTR) of HIV-1 transcripts is targeted by microRNA 28 (miR-28), miR-125b, miR-150, miR-223, and miR-382, which are reduced during monocyte-to-macrophage differentiation (30). Among these microRNAs, miR-28, miR-150, miR-223, and miR-382 are induced in IFN-α-activated, HIV-resistant MDMs (28). miR-155 targets the HIV dependency factors ADAM10, TNPO3, NUP153, and LEDGF/p75 in Toll-like receptor 3 (TLR3) ligand-treated MDMs (31). Importantly, microRNAs and other regulatory RNAs modulate viral entry. Kulkarni et al. (32) recently showed that the antisense long noncoding RNA (lncRNA) CCR5AS inhibits CCR5 mRNA degradation mediated by the RNA-binding protein RALY in CD4^+^ T cells. We previously reported that TNF-α-induced miR-221 and miR-222 target CD4 mRNAs in MDMs (24). Thus, variations in CCR5AS and miR-221/222 lead to differences in CCR5 and CD4 expression, respectively, directly impacting viral entry.

Following comprehensive analyses of transcriptome sequencing (RNAseq) microRNA expression profiles in HIV-1-infected MDMs, HIV-1-exposed but noninfected bystander MDMs, and virus-unexposed control MDMs (24), we identified two closely related p53-regulated microRNAs, miR-103 and miR-107, as negative regulators of CCR5, which are enhanced in bystander cells. Functional analyses of miR-103 reveal that it is induced by interleukin-1β (IL-1β) and modulates MDM permissiveness to HIV-1 by inhibiting viral entry via CCR5 downregulation. Importantly, we found that miR-103 and miR-107 are upregulated in colon macrophages as well as in macrophages recovered by bronchoalveolar lavage (BAL) from HIV-infected individuals under ART, thus highlighting the role of these microRNAs in regulating HIV-1 entry in these tissue-resident macrophages.

## RESULTS

### CCR5 mRNA is a target of miR-103 and miR-107 in MDMs.

**FIG 1:**
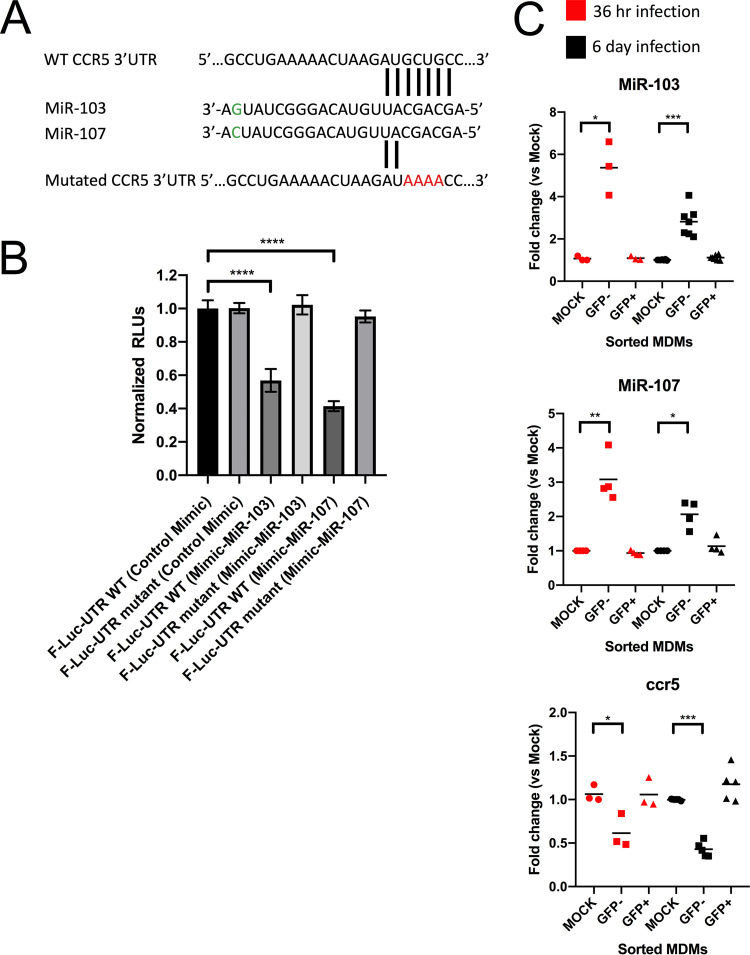
miR-103 and miR-107 are enhanced and CCR5 is downregulated in bystander MDMs. (A) Nucleotide sequences of miR-103 and miR-107 showing the matching seed sequence nucleotides recognized in the CCR5 mRNA 3′ UTR. The nucleotides changed in the mutated version used in the target validation assay are in red. The single G/C substitution in miR-103 compared to miR-107 is shown in green. The CCR5 nucleotide sequence is from GenBank accession number NM_000579. (B) Target validation assay in wild-type (WT) or mutant firefly luciferase (F-Luc)-CCR5-3′-UTR-transfected HEK293T cells treated with either control, miR-103, or miR-107 mimics. Results are plotted as mean F-Luc activities (relative light units [RLUs]) standardized to the control *Renilla* Luc activity ± standard deviations (SD). Statistical significance was determined using Student’s *t* test (*n* = 4 technical replicates). (C) miR-103, miR-107, and CCR5 expression levels were determined in sorted macrophages derived from 3 to 7 blood donors by real-time qPCR. Bars shown are the mean fold changes compared to mock cells (*n* = 3 to 7 blood donors; Wilcoxon matched-pairs signed-rank test).

**FIG 2:**
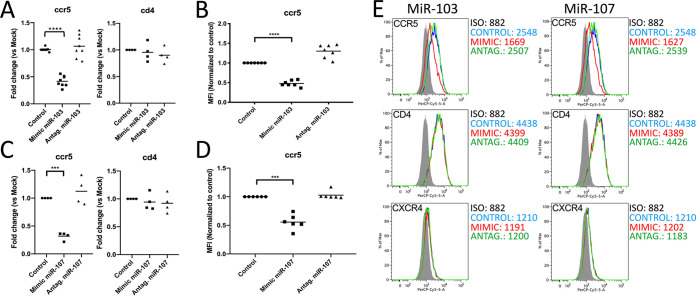
Effect of transfected miR-103 or miR-107 mimics, or antagomirs, on CCR5 expression in MDMs. MDMs from 4 to 8 blood donors were transfected with either the indicated controls, mimics, or antagomirs and processed accordingly. (A) CCR5 and CD4 mRNA levels were quantified by real-time qPCR in control, miR-103 mimic-transfected, or miR-103 antagomir-transfected MDMs and compared to those in control-transfected cells. Bars are the mean fold changes compared to the control (*n* = 4 to 8 blood donors; Wilcoxon matched-pairs signed-rank test). (B) Surface CCR5 was measured by flow cytometry in control, miR-103 mimic-transfected, or miR-103 antagomir-transfected MDMs, and the geometric mean fluorescence intensity (MFI) was calculated relative to control-transfected cells. Bars are the mean relative MFIs (*n* = 7 blood donors; Wilcoxon matched-pairs signed-rank test). (C) CCR5 and CD4 mRNA levels were quantified by real-time qPCR in control, miR-107 mimic-transfected, or miR-107 antagomir-transfected MDMs and compared to those in control-transfected cells. Bars are the mean fold changes compared to the control (*n* = 4 blood donors; Wilcoxon matched-pairs signed-rank test). (D) Surface CCR5 was measured by flow cytometry in control-, miR-107 mimic-, or miR-107 antagomir-transfected MDMs, and the MFI was calculated relative to control-transfected cells. Bars are the mean relative MFIs (*n* = 6 blood donors; Wilcoxon matched-pairs signed-rank test). (E) Representative flow cytometry data for MDMs from one blood donor shown in panels B and D. Additional surface expression levels (MFIs) for CD4 and CXCR4 are also shown. ISO, isotype control.

In order to identify new microRNAs that modulate the susceptibility of macrophages to HIV infection, we screened a microRNA expression database that we previously generated by RNAseq deep sequencing of uninfected MDMs as well as productively infected (green fluorescent protein-positive [GFP^+^]) and bystander (GFP-negative [GFP^−^]) MDMs infected with a GFP reporter virus (24). As performed previously, we limited our screen to the 30 most highly expressed microRNAs that were upregulated in bystander macrophages after 6 days of HIV-1 infection (see Table S1 in the supplemental material) but slightly expanded our search to include those with less striking increases. Using this approach, we found that microRNA 103 (log_2_ = 0.31; *P* = 0.065; ranked 23rd) was enhanced in GFP-negative MDMs. miR-103 was selected given that a closely related microRNA, miR-107, was also slightly upregulated in bystanders (log_2_ = 0.19), although it was not among the 30 most highly expressed microRNAs (ranked 87th out of 414). Indeed, we confirmed that miR-107 was expressed (∼400 molecules/cell) about 5-fold less than miR-103 (∼2,000 molecules/cell) in uninfected MDMs by serial dilution quantitative PCR (qPCR). Interestingly, an ensuing search for the most relevant targets of miR-103 and miR-107 using the Targetscan database (www.targetscan.org) revealed that the HIV-1 entry coreceptor CCR5 contains a potential miR-103/107 target site in its 3′ UTR (Fig. 1A). To test CCR5 as a potential target for miR-103/107, we first validated whether the 3′ UTR of CCR5 could be a target of both miR-103 and miR-107 in a reporter assay in which mRNA of the 3′ UTR of CCR5 fused to that of firefly luciferase (F-Luc) is expressed in either control-, miR-103 mimic-, or miR-107 mimic-transfected HEK293T cells. The presence of the miR-103 or miR-107 mimic significantly reduced Luc expression, and this was not observed in cells expressing a CCR5 3′ UTR mutated in the seed sequence (Fig. 1B). The enhanced expression of both miR-103 and miR-107 in bystander MDMs was validated by sensitive two-tailed reverse transcription-qPCR (qRT-PCR) (33) in GFP-sorted MDMs obtained from 3 to 7 donors at either 36 h or 6 days of HIV-1 infection (Fig. 1C). Accordingly, we determined that CCR5 mRNAs levels were significantly reduced in GFP-negative bystander MDMs compared to uninfected cells (Fig. 1C). Finally, the expression of CCR5 was also monitored directly in MDMs transfected with mimics or antagomirs for miR-103 (7 to 8 donors) or miR-107 (4 to 6 donors), respectively, and compared to that of the controls (Fig. 2). A >2-fold reduction in CCR5 mRNA and an ∼2-fold reduction in CCR5 cell surface expression were observed in either miR-103 or miR-107 mimic-transfected cells. Interestingly, no significant difference in CCR5 mRNA or cell surface expression was detected in either miR-103 or miR-107 antagomir-transfected MDMs and control cells, suggesting that at steady state, these microRNAs do not limit CCR5 expression (Fig. 2).

10.1128/mBio.02314-20.3 TABLE S1 List of the 30 most highly expressed microRNAs in MDMs. (Based on data from reference 24.) Download Table S1, PDF file, 0.04 MB. Copyright © 2020 Lodge et al. 2020 Lodge et al. This content is distributed under the terms of the Creative Commons Attribution 4.0 International license.

### miR-103 mimics restrict HIV replication in macrophages by targeting CCR5-mediated viral entry.

**FIG 3:**
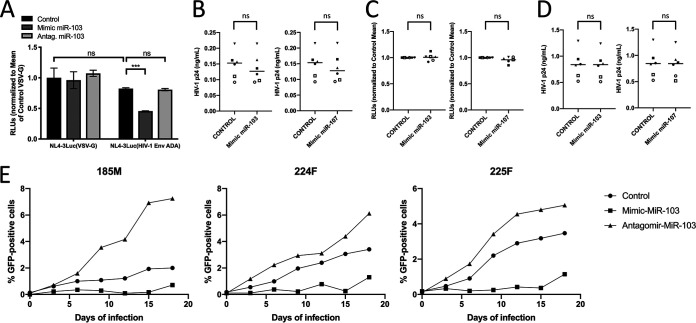
Effect of transfected miR-103 mimics or antagomirs on HIV-1 infection in MDMs. (A) MDMs isolated from 4 blood donors were treated with the indicated mimics or antagomirs, or controls, and infected with HIV-1 Env (ADA)- or VSV-G-pseudotyped luciferase-encoding HIV-1, and F-Luc activity was determined in the lysates 48 h following infection. RLUs were normalized to the value for F-Luc obtained in the VSV-G-pseudotyped virus-infected cells. Mean relative F-Luc activities ± SD are shown (*n* = 4 blood donors; Student’s *t* test). ns, not significant. (B) MDMs from 6 blood donors (represented by different symbols) were transfected with either controls or the indicated mimics and infected with VSV-G-pseudotyped CXCR4-tropic NL4-3. The level of HIV-1 released into the supernatant was determined after 4 days of infection by an ELISA for HIV-1 p24 (*n* = 6 blood donors; Student’s *t* test). (C) The infectivity of the released viruses in panel B was measured by determining F-Luc activity in infected TZM-bl reporter cells (*n* = 6 blood donors; Student’s *t* test). (D) Same as for panel B except that a VSV-G-pseudotyped NL4-3ΔEnv virus was used. (E) MDMs from 3 donors were transfected with either the control, the miR-103 antagomir, or the miR-103 mimic and infected with GFP-expressing HIV-1. The percentage of GFP-positive cells was determined at the indicated time points.

Since variation in CCR5 levels impacts the susceptibility of macrophages to HIV-1 (34), we tested if miR-103-mediated CCR5 decline affects HIV-1 infection. We focused mostly on miR-103 given its high levels in macrophages compared to miR-107. We compared Luc activities in MDMs infected with single-round Luc reporter viruses coated with either HIV-1 ADA Env (CCR5-tropic) or vesicular stomatitis virus G (VSV-G) (CD4/CCR5-independent) glycoproteins (Fig. 3A). The transfection of miR-103 mimics significantly inhibited Luc activity in MDMs infected with HIV-1(ADA Env) but not in those infected with HIV-1(VSV-G) (Fig. 3A). We observed no significant difference in Luc signals of MDMs infected with VSV-G-coated virus between control and mimic-transfected cells, suggesting that miR-103 mimics primarily affect CCR5-dependent viral entry. Consistent with the lack of an effect of miR-103 on CCR5 at steady state, transfection of miR-103 antagomirs did not impact CCR5-dependent viral entry. In order to investigate if either miR-103 or miR-107 influenced steps other than entry in MDMs, mimic-treated macrophages were infected with either CXCR4-tropic (Fig. 3B and C) or Env-negative (fig. 3D) HIV-1 that was VSV-G pseudotyped, thus limiting the analysis to a single round of infection. Similar levels of virus, as measured by an HIV-1 p24 enzyme-linked immunosorbent assay (ELISA), were detected in control and mimic-treated cell supernatants (Supnts) (Fig. 3B and D), and no change in the infectivity of the resulting CXCR4-tropic viruses was detected in TZM-bl reporter cells (Fig. 3C). These results, combined with those obtained with VSV-G-pseudotyped HIV particles, indicate that miR-103 and miR-107 do not target any other steps in HIV-1 replication besides CCR5-mediated entry in MDMs.

To further assess the influence of miR-103 on HIV-1 replication kinetics in MDMs, we monitored viral spread in macrophages infected with a GFP-expressing virus (3 donors) (Fig. 3E). In all cases, transfection of miR-103 mimics prior to the initial HIV-1 input reduced the frequency of GFP-expressing MDMs over 3 weeks (Fig. 3E). Interestingly, transfection of miR-103 antagomirs in this experimental setting enhanced the spread of HIV-1 infection in MDMs from all 3 donors, culminating in an additional (1.3- to 3-fold) increase in GFP-expressing MDMs after 3 weeks (Fig. 3E).

### HIV-1-infected MDMs produce miR-103-enhancing cytokines.

**FIG 4:**
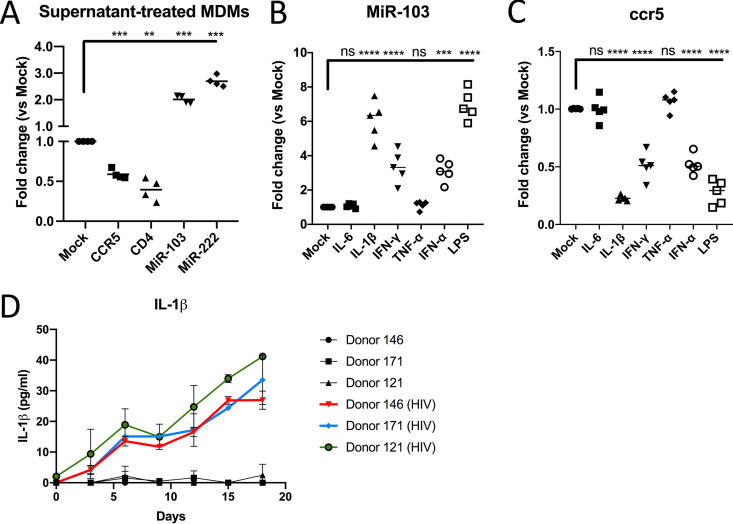
Macrophage activation by IL-1β reduces CCR5 expression and enhances miR-103 (see also Fig. S1 in the supplemental material). (A) Virus-cleared supernatants from infected macrophages were added to new MDM cultures (*n* = 4 blood donors; Wilcoxon matched-pairs signed-rank test) for 48 h, and the levels of CCR5 mRNA, CD4 mRNA, miR-103, and miR-222 were determined by real-time qPCR. Bars represent the mean fold changes compared to mock (untreated) cells. (B) MDMs derived from different blood donors (*n* = 5; Wilcoxon matched-pairs signed-rank test) were treated with the indicated MDM activators for 48 h, and miR-103 expression was measured by real-time qPCR. Bars represent the mean fold changes compared to mock (untreated) cells. (C) CCR5 mRNA levels were measured by real-time qPCR in the same samples as the ones described above for panel B. Bars represent the mean fold changes compared to mock (untreated) cells. (D) MDMs from 3 blood donors were infected with HIV-1, and the levels of IL-1β released into the supernatants were compared to that of uninfected cells at the indicated time points by an ELISA. Shown are picograms per milliliter ± SD of IL-1β.

The activity of the miR-103 antagomirs on HIV-1 infection kinetics led us to hypothesize that spreading HIV-1 infection might induce the secretion of soluble factors in MDM cultures. These factors would induce an upregulation of miR-103 expression levels functionally blockable by antagomirs. Indeed, such levels are not obtained in uninfected MDMs (Fig. 2) or in single-round infections (Fig. 3A), reminiscent of our previous observations on the targeting of the CD4 receptor by miR-221 and miR-222 (24). We thus examined the effect of virus-cleared supernatants of 3-day HIV-infected macrophage cultures on the expression of miR-103 and CCR5 in naive MDMs (4 donors) (Fig. 4A). Macrophages treated with these conditioned Supnts expressed significantly reduced levels of CCR5 mRNA and, accordingly, 2-fold-increased miR-103 expression (Fig. 4A). As expected, Supnt-treated MDMs also showed reduced levels of CD4 mRNA and enhanced miR-222 (24). In order to identify possible secreted factors required for increased miR-103 expression, a panel of cytokines and macrophage activators was tested for miR-103 (5 donors) and CCR5 mRNA (5 donors) levels (Fig. 4B and C). Although most tested factors enhanced miR-103, the strongest increase observed was that by lipopolysaccharide (LPS) or IL-1β treatment, which also resulted in the greatest reduction of CCR5 mRNA in MDMs (Fig. 4B and C). Transfection of miR-103 antagomirs significantly (3 donors) increased CCR5 (but not control CD4) mRNA in either LPS-, IL-1β-, or Supnt-treated, but not in IFN-γ- or IFN-α-treated, MDMs. This suggests that the decrease in CCR5 mRNA triggered by IL-1β or LPS, an inducer of IL-1β (35, 36), is at least in part due to miR-103, although antagomirs have a transfection efficiency close to 60% (24). We also determined by ELISAs that IL-1β is secreted in HIV-1-infected macrophage cultures (Fig. 4D), a finding consistent with miR-103 and miR-107 accumulation in bystander MDMs.

10.1128/mBio.02314-20.1 FIG S1 miR-103 antagomirs counteract IL-1β-driven CCR5 reduction (Fig. 4). MDMs from 3 blood donors were treated with conditioned medium (Fig. 4A) or the indicated MDM activators (Fig. 4B and C) and then transfected with either control or miR-103 antagomirs. CCR5 or CD4 mRNA expression levels were measured by real-time qPCR. Bars represent the mean fold changes compared to mock (untreated) cells (*n* = 3 blood donors; Wilcoxon matched-pairs signed-rank test). Download FIG S1, PDF file, 0.1 MB. Copyright © 2020 Lodge et al. 2020 Lodge et al. This content is distributed under the terms of the Creative Commons Attribution 4.0 International license.

**FIG 5:**
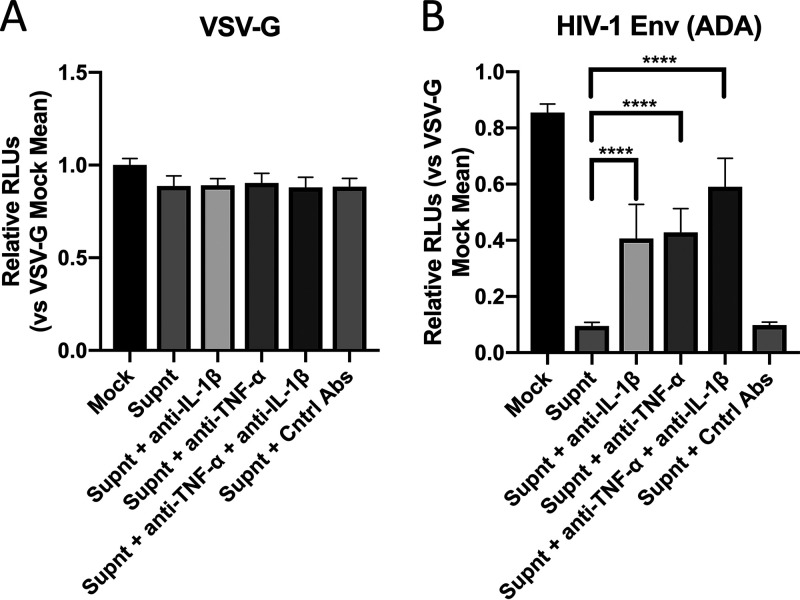
IL-1β and/or TNF-α secreted into HIV-1-infected MDM supernatants inhibits CCR5/CD4-dependent HIV-1 infection of macrophages (see also Fig. S2 in the supplemental material). The virus-cleared supernatant from infected macrophages was added to new MDM cultures from 3 blood donors, prior to infection with the indicated F-Luc-encoding viruses (pseudotyped with VSV-G [A] or HIV-1 Env ADA [B]), and F-Luc activity in the cell lysates was measured. In some cases, the conditioned supernatant was treated with either control goat anti-human IgGs (Cntrl Abs), neutralizing anti-TNF-α, neutralizing anti-IL-1β, or both neutralizing antibodies. Mean F-Luc activities ± SD are shown (*n* = 3 blood donors; Student’s *t* test).

Finally, to directly establish the role of IL-1β in modulating CCR5 expression via miR-103 in the context of HIV-1 infection, we pretreated MDMs with the Supnt alone or in the presence of either neutralizing anti-IL-1β, anti-TNF-α, or a combination of both antibodies (Abs). Neutralizing TNF-α was used given that TNF-α decreases CD4 and CD4-mediated HIV-1 entry via miR-221/222 (24). These cells were then infected with the single-cycle luciferase reporter viruses (Fig. 3A). As expected, the CCR5 HIV-1 Env-tropic luciferase viruses poorly infected Supnt-treated macrophages; however, this inhibition was partly alleviated by the presence of neutralizing anti-IL-1β or anti-TNF-α Ab (Fig. 5). Treatment of the Supnt with both Abs further restored the susceptibility of MDMs to CCR5 HIV-1 Env-tropic virus infection, thus underlining the antiviral impact of IL-1β and TNF-α. No effect was observed using CD4/CCR5-independent VSV-G-coated viruses, strongly suggesting that the supernatant containing IL-1β was inhibiting viral entry in part by decreasing the mRNA levels of CCR5 (Fig. 5A). We further confirmed this by measuring the levels of miR-103 in MDMs treated with the Supnt with or without the neutralizing Abs; indeed, a decrease in miR-103 was detected specifically in anti-IL-1β Ab-treated cells (Fig. S2). These results support a role of IL-1β in the induction of miR-103 and the downregulation of CCR5 during HIV-1 infection of MDMs, yet other factors secreted into the supernatant, such as IFN-α (37) (Fig. 4 and Fig. S1), RANTES, macrophage inflammatory protein 1α (MIP-1α), and MIP-1β (38), are also likely to contribute to the reduced levels of CCR5 (at both the mRNA and surface protein levels) in Supnt-exposed cells.

10.1128/mBio.02314-20.2 FIG S2 IL-1β secreted into HIV-1-infected MDM supernatants enhances miR-103 in MDMs (Fig. 5). The conditioned supernatant from infected macrophages was added to new MDM cultures from 4 blood donors. In some cases, the conditioned supernatant was treated with either control goat anti-human IgGs (cntrl Abs), neutralizing anti-TNF-α, neutralizing anti-IL-1β, or both neutralizing antibodies. miR-103 and miR-222 expression levels were then measured by real-time qPCR. Bars represent the mean fold changes compared to mock (untreated) cells (*n* = 4 blood donors; Wilcoxon matched-pairs signed-rank test). Download FIG S2, PDF file, 0.2 MB. Copyright © 2020 Lodge et al. 2020 Lodge et al. This content is distributed under the terms of the Creative Commons Attribution 4.0 International license.

### IL-1β restricts HIV-1 entry in MDMs by p53-induced miR-103.

**FIG 6:**
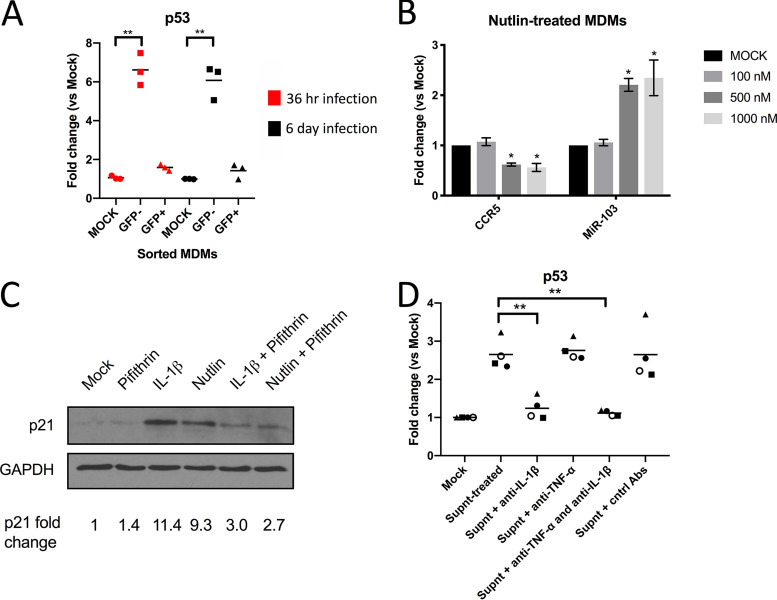
IL-1β-triggered p53 activity modulates CCR5 mRNA and miR-103 levels. (A) Expression of p53 mRNA was determined in sorted macrophages derived from 3 blood donors by real-time qPCR. Bars shown are the mean fold changes compared to mock cells (*n* = 3 blood donors; Wilcoxon matched-pairs signed-rank test). (B) Different concentrations of the MDM2 antagonist Nutlin-3 were added to MDMs (3 different blood donors), and its effect on CCR5 mRNA and miR-103 levels was determined by real-time qPCR. Shown are the mean fold changes ± SD compared to mock cells (*n* = 3 blood donors; Student’s *t* test). (C) Control, IL-1β-treated, or Nutlin-treated MDMs were then exposed to Pifithrin (or the vehicle) for 24 h and lysed, and the p53-driven enhanced expression of p21 was determined by Western blotting. Data shown are derived from MDMs of one blood donor (*n* = 2 blood donors). (D) The conditioned supernatant from infected macrophages was added to new MDM cultures from 4 blood donors. In some cases, the conditioned supernatant was treated with either control goat anti-human IgGs (Cntrl Abs), neutralizing anti-TNF-α, neutralizing anti-IL-1β, or both neutralizing antibodies. The level of p53 mRNA was then measured by real-time qPCR. Bars represent the mean fold changes compared to mock (untreated) cells (*n* = 4 blood donors; Wilcoxon matched-pairs signed-rank test).

IL-1β has been reported to activate p53-dependent pathways (39), and both miR-103 and miR-107 have been shown to be upmodulated by p53 (40–42). Since miR-103 and miR-107 are upregulated in bystander MDMs (Fig. 1), we confirmed that p53 mRNA is also enhanced in these cells (Fig. 6A) following either 36 h or 6 days of HIV-1 infection. Furthermore, Nutlin-3, an MDM2 antagonist and p53 pathway activator (43), increased miR-103 levels in treated MDMs, leading to a decrease in CCR5 mRNA (Fig. 6B), thus validating the regulation of miR-103 by p53. We confirmed the enhanced activity of p53 in either IL-1β- or Nutlin-treated MDMs by showing an increase of p21, a well-established p53-regulated protein, in these cells, which was inhibited by the p53 inhibitor Pifithrin (fig. 6C). Importantly, Supnt treatment enhanced p53 mRNA in MDMs, and this increase was completely abolished by neutralizing anti-IL-1β Abs (Fig. 6D).

**FIG 7:**
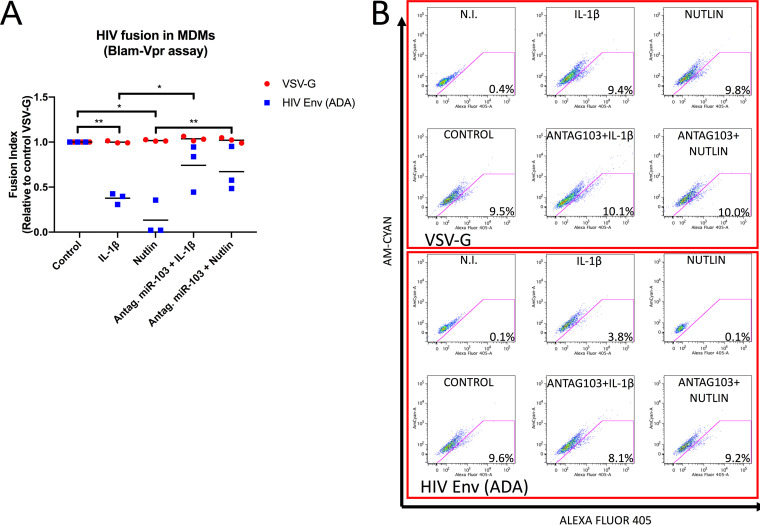
IL-1β triggers p53-mediated inhibition of CCR5-dependent HIV-1 entry in macrophages through miR-103. MDMs derived from different blood donors (*n* = 3) were treated with the indicated reagents for 48 h and then left untreated or additionally treated with miR-103 antagomirs. (A) Macrophages were then infected with either the VSV-G- or HIV-Env (ADA)-pseudotyped Blam-Vpr-containing viruses. Bars represent mean viral fusion efficiencies compared to VSV-G-pseudotyped virus fusion in control-treated cells (“fusion index”). Statistics were calculated by Wilcoxon matched-pairs signed-rank tests. (B) Data from a representative experiment (numbers represent percentages of Alexa Fluor 405-positive cells). N.I., noninfected control.

To specifically test the effect of the IL-1β-mediated induction of miR-103 on HIV-1 entry in MDMs, we used the Blam-Vpr fusion assay (44). Compared to VSV-G-pseudotyped control viruses, the fusion step of HIV-1 CCR5-tropic Env-coated viral particles was strongly inhibited by either IL-1β or Nutlin-3. Importantly, these inhibitory effects were substantially abolished by treatment with miR-103 antagomirs (Fig. 7), indicating that inhibition of viral entry by IL-1β in macrophages involves p53-regulated miR-103 and, most likely, miR-107.

### HIV-resistant tissue-resident macrophages express high levels of miR-103/107.

**FIG 8:**
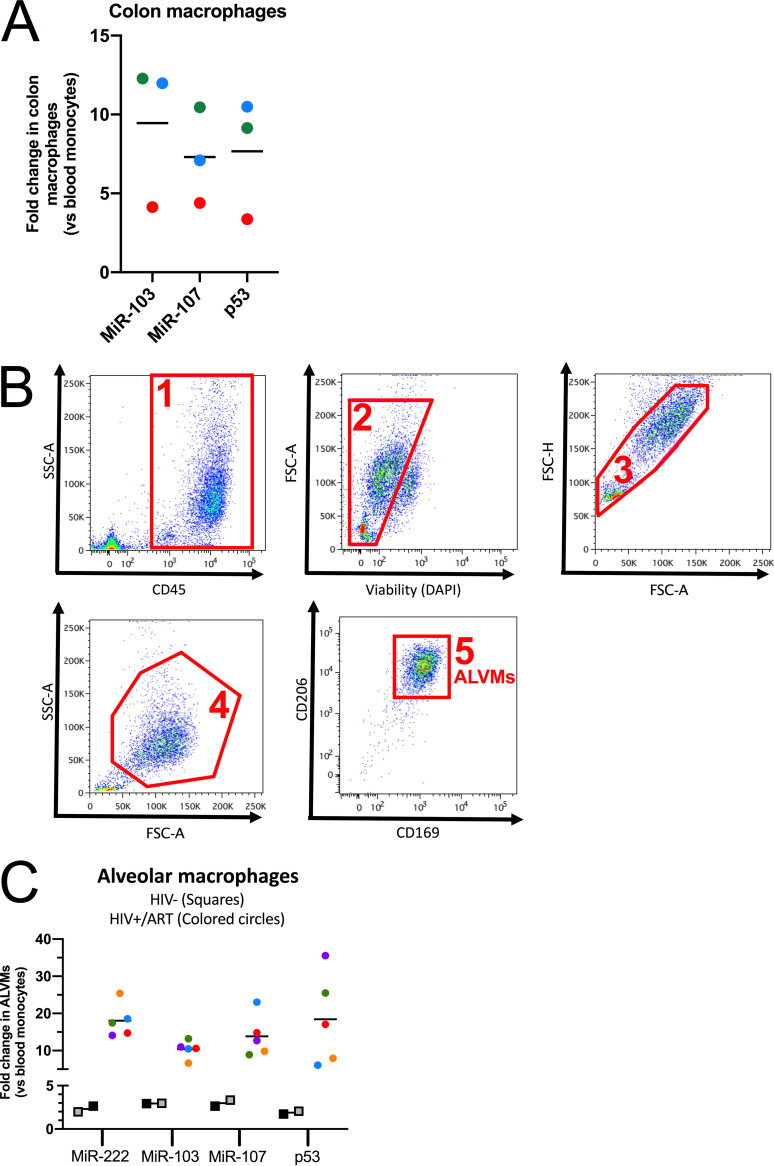
HIV-resistant tissue-resident macrophages express high levels of miR-103/107. (A) Myeloid cells were sorted from matched sigmoid intestine biopsy specimens and blood of 3 HIV-negative study participants. The expression levels of miR-103, miR-107, and p53 mRNA in blood and colon myeloid cells were determined by real-time qPCR and normalized to the levels in blood myeloid cells (*n* = 3 donors, represented by different colors). (B) Fluorescence-activated cell sorting (FACS) strategy (steps 1 to 5) to obtain lung alveolar macrophages (ALVMs). DAPI, 4′,6-diamidino-2-phenylindole. SSC-A, side scatter (area); FSC-A, forward scatter (area); FSC-H, forward scatter (height). (C) ALVMs and blood monocytes were obtained from 5 HIV-positive (under ART) or 2 HIV-negative individuals, and the levels of p53 mRNA, miR-222, miR-103, and miR-107 were determined by real-time qPCR. RNA levels of ALVMs were normalized to that of the blood monocytes (*n* = 2 or 5 donors, represented by different colors). In all cases, bars represent the mean fold changes compared to the levels in blood myeloid cells. *P* values for fold changes are all <0.01 (**) compared to blood myeloid cells, from Wilcoxon matched-pairs signed-rank tests. Each dot represents a participant.

To ascertain a more physiological sense of miR-103/107 in tissue-resident macrophages, we examined the levels of miR-103, miR-107, and p53 mRNA in colon macrophages obtained from healthy donors (24) as well as in lung alveolar macrophages (ALVMs) recovered from either HIV-negative or HIV-infected individuals under long-term antiretroviral therapy (>9 years) (45, 46) and compared their expression levels to those in blood monocytes obtained from the same individuals. First, as we previously described for the CD4-targeting miR-221 (24), miR-103/107 and p53 mRNA were greatly upregulated in colon macrophages (3 donors) (Fig. 8A), thus potentially contributing to the resistance of these cells to HIV-1 infection (22, 23). In alveolar macrophages, which were isolated as described in the legend of Fig. 8B, the levels of miR-103/107, p53 mRNA, as well as miR-222 were slightly increased, compared to those in blood monocytes, in HIV-1-negative participants (*n* = 2 donors) (Fig. 8C) but were highly upregulated in aviremic HIV-infected individuals (*n* = 5 donors) (Fig. 8C), suggesting that in the latter context, local environmental inflammatory signals may drive ALVMs to an antiviral state that likely limits HIV-1 entry and infection via the upregulation of microRNAs, including miR-103/107 and miR-221/222.

## DISCUSSION

Levels of chemokine receptor CCR5 expression have a central role in determining the risk of HIV-1 acquisition and disease progression (3). Deciphering the multiple facets of regulation that condition CCR5 expression in HIV-1 target cells is therefore needed to better understand the influence of CCR5 on the outcome of HIV-1 infection and for advancing the development of CCR5-targeted therapies. In this study, we took advantage of a previous profiling of microRNAs differentially expressed in HIV-1-infected and virus-exposed noninfected MDMs that had identified miR-221 and miR-222 as modulators of CD4 expression (24), to search for microRNAs regulating CCR5 expression in macrophages. Indeed, the levels of CCR5 mRNA in virus-exposed, noninfected bystander MDMs were downregulated, leading us to focus on upregulated microRNAs in these cells that would potentially target the 3′ UTR of CCR5 and limit HIV-1 entry.

Among the microRNAs that were upregulated in bystander MDMs, two closely related microRNAs, miR-103 and miR-107, were identified as modulators of CCR5 expression. These two paralogs reside on different human chromosomes and are encoded within introns in the genes that encode pantothenate kinase (PANK) enzymes that are essential for the biosynthesis of coenzyme A (47). The expression of miR-103/107 has been shown to regulate systemic glucose metabolism and insulin sensitivity (48) and has been associated with many types of cancers, including colorectal cancers (49, 50). Our data reveal that miR-103/107 upregulation in MDMs significantly reduces CCR5 mRNA and surface protein expression levels and as a result downregulates HIV-1 entry. Despite being able to target the expression of multiple host proteins involved in cell growth, motility, and adhesion (CDK6, P130, LATS2, DAPK, KLF4, and Axin) (42, 49, 50), survival (LRP) (41), and carbohydrate and fatty acid metabolism (caveolin-1) (47, 48, 51), the expression of miR-103/107 mimics had no detectable effects on VSV-G-pseudotyped HIV-1 infection or on late stages of the virus replication cycle that govern the production of infectious virus particles. These findings demonstrate that in the context of macrophages, these microRNAs impact specifically HIV-1 entry by interfering with Env-mediated viral fusion via CCR5. Interestingly, although miR-103/107 antagomirs had no effect on CCR5 expression in uninfected MDMs, these antagomirs alleviated CCR5 downregulation under conditions in which the expression of miR-103/107 was induced by cytokine treatment, notably IL-1β. This observation is reminiscent of the disrupting effect of miR-221/222 antagomirs on TNF-α-mediated CD4 downregulation via miR-221/222 upregulation (24). Taking this into account, the presence of miR-103/107 antagomirs in the context of HIV-infected MDMs was shown to significantly enhance the spread of HIV-1 infection, validating that miR-103/107 expression levels are increased in noninfected bystander cells, thus making them refractory to HIV-1 infection.

Infection of MDMs with HIV-1 *in vitro* results in the upregulation and constitutive secretion of IL-1β in culture supernatants (52). Our data demonstrate that the release of IL-1β by infected macrophages induces an upregulation of miR-103 in MDMs treated with a conditioned supernatant, a state that leads to a reduction of CCR5 mRNA levels. Although the exposure of MDMs to other cytokines reported to inhibit HIV-1 replication in macrophages, such as IFN-γ (27) and IFN-α (53, 54), downregulated CCR5 mRNA, this reduction was not affected by miR-103 antagomirs, thus excluding a direct role of miR-103 in IFN-γ- or IFN-α-mediated downmodulation of CCR5 expression levels. However, the addition of the miR-103 antagomirs did not result in a complete recovery of CCR5 levels in conditioned supernatant- or IL-1β-treated MDMs, suggesting that other factors/cytokines participate in CCR5 modulation through an independent process. IL-1β binding to IL-1β receptor 1 was reported to stimulate HIV-1 transcription through a mechanism independent of the activation of NF-κB (55). However, it was also shown that IL-1β can inhibit HIV-1 entry by downregulating the CD4 receptor at the transcriptional level (24, 36). Taken together, our results further extend these findings and provide direct evidence that IL-1β can also inhibit HIV entry by disrupting HIV-1 Env-mediated fusion by triggering miR-103/107-directed downmodulation of CCR5 mRNAs. That being said, it is important to note that innate immune activation of macrophages occurs beyond IL-1β and affects a broad array of transcriptional factors that can affect viral restriction at different stages of the virus replication cycle. Indeed, a recent paper by Covino et al. (56) shows how the activation of an NF-κB/miR-155 regulatory network upon the neutralization of CCL2 contributes to the restriction of HIV-1 replication in macrophages at postentry steps, notably through APOBEC3A.

Previous reports have shown that miR-103/107 expression is regulated by p53 (40–42). Furthermore, besides its tumor suppressor function, p53 has been found to modulate the macrophage response to environmental challenges and regulate proinflammatory gene responses in these cells (57–59). Thus, our finding that the regulation of CCR5 expression by IL-1β is at least in part triggered by p53-modulated microRNAs reinforces the notion that p53 plays a key role in integrating environmental signals and, more specifically, an inflammatory signal triggered by IL-1β and highlights how the resulting p53-modulated response impacts the permissiveness of macrophages to HIV-1 infection. Indeed, we show that p53 mRNA is enhanced in sorted bystander MDMs and that treatment of macrophages with the p53 stabilizer Nutlin-3 leads to inhibition of HIV-1 fusion through enhanced expression of miR-103/107 and reduction of CCR5 expression. Given the multifunctional nature of p53, it is conceivable that other steps of the HIV-1 replicative cycle, downstream of viral entry, may also be affected upon p53 stabilization and activation. Indeed, we recently showed that downmodulation of MDM2, the E3 ubiquitin ligase responsible for p53 degradation, impairs early HIV-1 postentry steps in macrophages (60), suggesting that p53 activation is restrictive for HIV-1 infection in MDMs. In that regard, the p53-dependent increase of p21 in macrophages was shown to enhance the antiviral form of SAMHD1, a restriction factor that disrupts the production of HIV-1 cDNA (61). Overall, our current results and previous findings on miR-221/222 (62) underscore a sophisticated antiviral response triggered by the proinflammatory cytokines IL-1β and TNF-α in MDMs that impedes HIV-1 entry via p53- and NF-κB-regulated microRNAs targeting CCR5 and CD4 expression.

The great variation in the HIV-1 susceptibility of macrophages is due to their different activation levels, origins, and tissue localizations (63). Indeed, several antiviral restriction factors are enhanced in activated MDMs and target various steps of the viral replicative cycle. Macrophages directly isolated from different tissues also show remarkable differences in their susceptibility to HIV-1 infection, with intestinal macrophages displaying nonpermissiveness to HIV-1 infection because of a downregulation of CD4 and CCR5 (15, 22). Interestingly, our results reveal that besides expressing important levels of CD4-targeting miR-221/222, these cells express significant quantities of miR-103 and miR-107 that could also contribute to their resistance to HIV infection. Furthermore, our data show that lung alveolar macrophages express high levels of miR-103/107, miR-222, and p53 mRNA in the context of HIV-1-infected individuals undergoing ART. These levels are strikingly higher than those found in HIV-1-negative participants, suggesting that these tissue-resident macrophages in ART-treated individuals might be in an activated state refractory to HIV-1 infection. Whether downregulation of CD4 and CCR5 expression by miR-221/222 and miR-103/107, respectively, represents one of the barriers for seeding the HIV-1 reservoir in these cells, perhaps explaining the varying occurrence and the low frequency of alveolar macrophages harboring proviral DNA in virally suppressed HIV-1-infected individuals (19, 46), awaits further studies.

**FIG 9:**
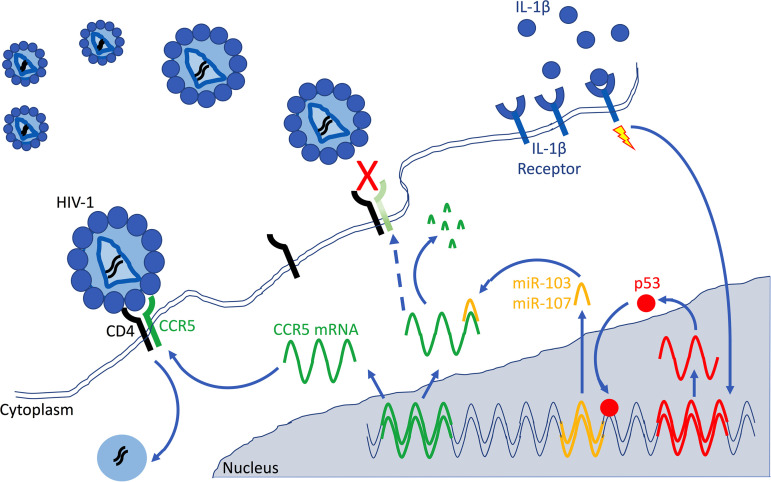
Model summarizing how miR-103 and miR-107 target CCR5 mRNA, leading to less CCR5-mediated HIV-1 entry in macrophages. The exposure of macrophages to the proinflammatory cytokine IL-1β (blue dots) triggers the expression of p53 (red dots). The resulting p53 response activates a set of p53-regulated genes, including miR-103 and miR-107 (yellow), which target CCR5 mRNAs (green) and downregulate their levels, presumably by inducing their degradation. The ensuing reduction of CCR5 expression at the cell surface (broken blue arrow and light green CCR5) limits HIV-1 entry and influences the permissiveness of macrophages to HIV-1 infection. This model highlights miR-103 and miR-107 as important p53-regulated effectors of the antiviral response triggered by the proinflammatory cytokine IL-1β in macrophages.

In conclusion, we have identified miR-103 and miR-107 as important p53-regulated effectors of the antiviral response triggered by the proinflammatory cytokine IL-1β, as summarized in Fig. 9. These microRNAs act as inhibitors of HIV-1 entry through their capacity to downregulate the CCR5 coreceptor. These findings raise the possibility that p53-activating drugs, many of which are currently in clinical trials, may have unforeseen effects on macrophage susceptibility to HIV-1 infection, thus influencing the establishment of an HIV-1 reservoir in these cells.

## MATERIALS AND METHODS

### Study subjects.

Peripheral blood samples were obtained from HIV- and hepatitis C virus (HCV)-seronegative adults (of either gender) or HIV-seropositive adults under suppressive ART in the case of human lung alveolar macrophages. Research protocols for the use of HIV-seronegative human blood cells were approved by the Research Ethics Review Board of the Institut de Recherches Cliniques de Montréal (IRCM). The study using sigmoid intestine biopsy specimens and blood from adult participants received approval from the Institutional Review Boards of the McGill University Health Centre and the Centre de Recherche du Centre Hospitalier de l’Université de Montréal (CR-CHUM) (64). The study using human lung alveolar macrophages and blood from participants received approval from the Institutional Review Boards of the McGill University Health Centre, the CR-CHUM, and the Université du Québec à Montréal (UQAM). All participants had given written informed consent, and all studies were performed in compliance with the Declaration of Helsinki.

### Plasmid constructs, CCR5 3′-UTR validation assay, and microRNA mimics and antagomirs.

The NL4-3 (65), NL4-3ΔEnv (66), NL4-3Env^−^Luc^+^Vpr^+^ (67), Blam-Vpr (44), SVCMV-VSV-G (62), SVIII-ADA-Env (68), NL4-3-ADA-IRES-GFP, and NL4-3Env^−^Vpr^−^ (24) constructs were previously described. For the miR-REPORT system (Applied Biosystems, Ambion), the plasmid pGL4.70Actin1.2(8) (69), encoding *Renilla* luciferase under the control of the actin promoter, was used instead of pMIR-REPORT-β-gal. In order to generate pMIR-REPORT-CCR5-Luc, blunt-end *Pfu* PCR was performed on cDNA of pooled primary peripheral blood lymphocytes (PBLs) using primers 5′CCR5UTR and 3′CCR5UTR (see Table S2 in the supplemental material), and the fragment equivalent to the full CCR5 3′ UTR was inserted into the SacI and blunted HindIII sites of pMIR-REPORT-Luc. Site-directed mutagenesis of the 3′ UTR of CCR5 was performed by 2-step overlap PCR using primers CCR5mutFwd and CCR5mutRev (Table S2) and cloned into pMIR-REPORT using the same strategy in order to generate pMIR-REPORT-CCR5mutLuc (in which the miR-103/miR-107 target sequence ATGCTGC is mutated to ATAAAAC). The 3′-UTR CCR5 target validation assay was performed as previously described (24) using pMIR-REPORT-CCR5-Luc or pMIR-REPORT-CCR5mut-Luc and the Dual-Glo luciferase assay system (Promega) on a GloMax luminometer (Promega). For absolute qRT-PCR analyses of miR-103 and miR-107, primers miR-103C-FWD and miR-103C-REV or primers miR-107C-FWD and miR-107C-REV were annealed and directly inserted into the EcoRI and BamHI sites of pEGFP-N1 to create pMIR-103 and pMIR-107, respectively. The miR-103-3p (catalog number YM00470828), miR-107 (catalog number YM00470827), and miR-222 (catalog number 472196-001) miRCURY locked nucleic acid (LNA) mimics or the miR-103-3p (catalog number YI04107448) and miR-107 (catalog number YI04109094) antagomirs were purchased from Qiagen (Exiqon). Nontargeting control RNAs were obtained from Dharmacon/GE Healthcare (siGENOME nontargeting 2, catalog number D-001210-02-20) or Ambion/Thermo Fisher Scientific (catalog number AM16104).

10.1128/mBio.02314-20.4 TABLE S2 Oligonucleotides used in this study. Download Table S2, PDF file, 0.1 MB. Copyright © 2020 Lodge et al. 2020 Lodge et al. This content is distributed under the terms of the Creative Commons Attribution 4.0 International license.

### Antibodies, flow cytometry, and chemicals used.

Antibodies used for flow cytometry assays were anti-human J418F1 or HEK/1/85a CCR5, 3.9 CD11c, 12G5 CXCR4, and OKT4 CD4 (all peridinin chlorophyll protein [PerCP]-Cy5.5); 3.9 CD11c-phycoerythrin (PE)-Cy7, M5E2 CD14-Pacific Blue, and 3G8 CD16-allophycocyanin (APC)-Cy7 were used with the corresponding isotype controls (BioLegend).

The Live/Dead fixable dead-cell stain kits were obtained from Life Technologies/Invitrogen. The following specific antibodies used to isolate colon myeloid cells were all from BioLegend: 9C4 CD326-Brilliant Violet 650 (BV650), RPAT8 CD8-PerCP-Cy5.5, HIB19 CD19-PerCP-Cy5.5, G10F5 CD66b-PerCP-Cy5.5, UCHT1 CD3-Alexa Fluor 700 or -Pacific Blue, and L243 HLA-DR-BV785. The specific antibodies used to isolate human alveolar macrophages were HI30 CD45-PE-Cy7, 19.2 CD206-PE, and 7-239 CD169-Brilliant Blue 515 (BB515) (all from BD Biosciences); OKT3 CD3-PE and HCD14 CD14-BV421 (both from BioLegend) were also used to isolate blood myeloid cells. Preparation of cells for flow cytometry and protein expression analyses was performed as previously described (24). The MDM2 inhibitor Nutlin-3 (Sigma) and the p53 inhibitor Pifithrin-α (EMD-Millipore) were used at 500 nM and 10 μM, respectively, unless mentioned otherwise, for 48 h.

### MDM isolation, activation, and transfection and HIV-1 production and infection.

MDMs were obtained from peripheral blood mononuclear cells (PBMCs) and characterized as previously described (24). MDM activation was induced by 48 h of treatment with the following reagents: LPS (100 ng/ml; Sigma), TNF-α (10 ng/ml; BioLegend), IL-6 (10 ng/ml; BioLegend), IFN-α (100 U/ml; PBL Interferon Source), IFN-γ (20 ng/ml; Peprotech), and IL-1β (10 ng/ml; BioLegend). Transfection of MDMs with either Exiqon microRNA LNA mimics or inhibitors (antagomirs) was performed using Lipofectamine RNAiMax (Invitrogen) as previously described (24). Following a 72-h incubation, cells were harvested for qRT-PCR or flow cytometry analyses. In some cases, a second transfection was performed. Viruses were produced and titers were determined as previously described (24), using the TZM-bl reporter cell line (70). Unless otherwise indicated, macrophages were infected with HIV-1 at a multiplicity of infection (MOI) of 1.

### SDS-PAGE and Western immunoblot analyses.

Sodium dodecyl sulfate-polyacrylamide gel electrophoresis (SDS-PAGE) of macrophage lysates and immunoblotting were performed as previously described (71) using rabbit anti-human 12D1 p21 (1/500; NEB/Cell Signaling) or mouse anti-glyceraldehyde-3-phosphate dehydrogenase (GAPDH) (BioLegend) diluted 1/200.

### Analysis of HIV-1 infection or fusion in MDMs, preparation of conditioned supernatants, and IL-1β measurement.

MDMs were transfected with either control RNA, mimics, or antagomirs and cultured for 72 h in 12-well plates. Cells were then infected with NL4-3-ADA-IRES-GFP virus at an MOI of 1, washed, and incubated for 2 weeks. Macrophages and their supernatants were harvested at the indicated time points, and viral spread was assessed by analyzing the percentage of cells expressing GFP by flow cytometry. For infected MDM conditioned supernatants, the supernatant was harvested at 48 h postinfection, ultracentrifuged on 20% sucrose cushions to clear it of virus particles, and then added (diluted 1/20) to new MDM cultures. Briefly, the conditioned supernatant was either left untreated, treated for 20 min at 37°C with neutralizing goat anti-human TNF-α or IL-1β monoclonal Abs (mAbs) (1 μg/ml; R&D Systems, Cedarlane) or control anti-human IgGs (1 μg/ml; Molecular Probes, Invitrogen), and added to macrophages 48 h prior to RNA extraction, the addition of antagomirs, or infection with VSV-G- or HIV-1 ADA-pseudotyped luciferase-encoding viruses. In the latter case, macrophages were lysed following 48 h of infection, and luciferase activity was determined. Virus production was assessed in some experiments by HIV-1 p24 ELISAs, and the infectivity of released virus was evaluated in TZM-bl reporter cells as described previously (24). Viral fusion was measured using the Blam-Vpr assay (44) as described previously (24). IL-1β in the supernatants from either control uninfected or infected MDMs was measured using an IL-1β ELISA Legend Max kit (BioLegend).

### RNA extraction, reverse transcription, and real-time qPCR analyses.

Total cellular RNAs were extracted using RNeasy RNA extraction columns (Qiagen) according to the manufacturer’s instructions and stored at −80°C. For the isolation of infected (GFP^+^) and bystander (GFP^−^) macrophage populations, MDMs infected with NL4-3-ADA-IRES-GFP viruses (MOI of 1) were sorted using an Influx cell sorter (BD Biosciences), and GFP^+^ and GFP^−^ cells were directly recovered in RLT lysis buffer as previously described (24). In the case of microRNAs, cDNAs were obtained by using two-tailed qRT-PCR (33); briefly, 100 to 300 ng of total RNAs was reversed transcribed using SuperScript II reverse transcriptase (Invitrogen) with poly(dT) and specific loop primers for the appropriate microRNAs. For qRT-PCR, cDNA and appropriate primers (Table S2) were added to SYBR green select master mix (Applied Biosystems) in 96-well plates and run on a ViiA96 thermocycler (Thermo Fisher Scientific). GAPDH was used as a loading control, and ΔΔ*C_T_* variations were calculated. For microRNA absolute quantitation, primers were designed according to methods described previously by Balcells et al. (72), and standardized dilutions of pMIR103 or pMIR107 were used as a reference to compute microRNA copy numbers. Particular attention was noted on melt curve analyses to obtain optimal conditions.

### Comparative expression of miR-103/107 and p53 mRNA in myeloid cells isolated from PBMCs or the colon.

Myeloid cells from either peripheral blood or sigmoid colon biopsy specimens were isolated from 3 adult HIV-negative donors, as previously described (23, 24). Cells stained with a cocktail of Abs were suspended in phosphate-buffered saline (PBS) with 5% fetal bovine serum (FBS) and 25 mmol/liter HEPES buffer, and the myeloid cell-enriched fraction with a CD326^−^ CD8^−^ CD19^−^ CD66b^−^ CD3^−^ HLA-DR^high^ phenotype was sorted on a BD-FACS Aria III instrument (BD Biosciences). The viability dye Live/Dead fixable aqua dead-cell stain kit (Invitrogen) was used to exclude dead cells. Sorted cells were lysed in RLT lysis buffer (Promega), and RNA was extracted for real-time qPCR.

### Comparative expression of miR-103/107, miR-222, and p53 mRNA in blood monocytes or ALVMs.

Individuals were recruited at the McGill University Health Centre and the CR-CHUM (Montreal, Canada). Bronchoalveolar lavage (BAL) specimens were collected from 2 HIV-negative individuals and 5 HIV-positive individuals under suppressive ART (undetectable plasma viral load and CD4 count higher than 350 cells/mm^3^) for at least 3 years and without any respiratory symptoms or active infections and processed as previously described (45). Matched peripheral blood was also collected from the participants, and CD14^+^ blood monocytes were isolated using an Influx cell sorter and recovered directly in RLT lysis buffer (Qiagen). BAL fluid cells were first washed and resuspended in a solution containing PBS–5% FBS–25 mM HEPES (pH 7.4), FcR blocked, and stained with a cocktail of Abs, and cells with the CD45^+^ CD206^+^ CD169^+^ phenotype were sorted. Live/Dead viability dye (Invitrogen) was used to exclude dead cells. Sorted cells were lysed in RLT lysis buffer, and RNA was extracted for mRNA or microRNA qRT-PCR.

### Statistics.

Statistical analyses were performed using the nonparametric Wilcoxon matched-pairs signed-rank test or Student’s *t* test. Statistical significance is indicated in the figures (*, *P* < 0.05; **, *P* < 0.01; ***, *P* < 0.001; ****, *P* < 0.0001).
