# Supplementary material for: Interleukin-1β Triggers p53-Mediated Downmodulation of CCR5 and HIV-1 Entry in Macrophages through MicroRNAs 103 and 107
Source: mBio. 2020 Sep 29;11(5):e02314-20. doi: 10.1128/mBio.02314-20 (PMC7527731; doi:10.1128/mBio.02314-20)
Supplement: TABLE S1 [file mBio.02314-20-st001.pdf]

Table S1: List of 30 highest expressed microRNAs in MDMs (based on data from ref. 24)

MicroRNA	Base mean average	Fold change GFP-neg./MOCK
hsa-miR-22-3p	1102115	0.93
hsa-miR-21-5p	845068	0.69
hsa-let-7f-5p	582709	1.12
hsa-miR-191-5p	505295	1.17
hsa-miR-146a-5p	389172	1.23
hsa-let-7a-5p	369882	1.30
hsa-miR-26a-5p	317539	0.99
hsa-miR-181a-5p	292114	1.13
hsa-miR-378a-3p	229967	1.07
hsa-let-7g-5p	149606	1.13
hsa-miR-146b-5p	139136	1.11
hsa-miR-222-3p	113630	1.82
hsa-miR-16-5p	113246	0.85
hsa-miR-30e-5p	96842	0.86
hsa-miR-142-5p	84684	0.62
hsa-miR-221-3p	75292	1.52
hsa-miR-26b-5p	71962	0.98
hsa-miR-423-3p	63087	1.69
hsa-miR-98-5p	61522	1.07
hsa-miR-186-5p	58768	1.08
hsa-miR-30d-5p	56068	0.98
hsa-miR-92a-3p	51372	1.07
hsa-miR-103a-3p	50807	1.24
hsa-miR-28-3p	43526	1.32
hsa-let-7d-5p	43158	1.32
hsa-let-7i-5p	40527	1.06
hsa-miR-342-3p	40451	1.43
hsa-miR-155-5p	39152	1.90
hsa-miR-29a-3p	34874	0.73
hsa-miR-30c-5p	32893	1.04

MiR-103 is shown in red. MiR-107 was ranked 87 out of the 414 microRNAs studied. MiR-221 and 222, that target the mRNA of the CD4 receptor, are highlighted in green.
