# Supplementary figures and images for: Interleukin-1β Triggers p53-Mediated Downmodulation of CCR5 and HIV-1 Entry in Macrophages through MicroRNAs 103 and 107
Source: mBio. 2020 Sep 29;11(5):e02314-20. doi: 10.1128/mBio.02314-20 (PMC7527731; doi:10.1128/mBio.02314-20)

ccr5

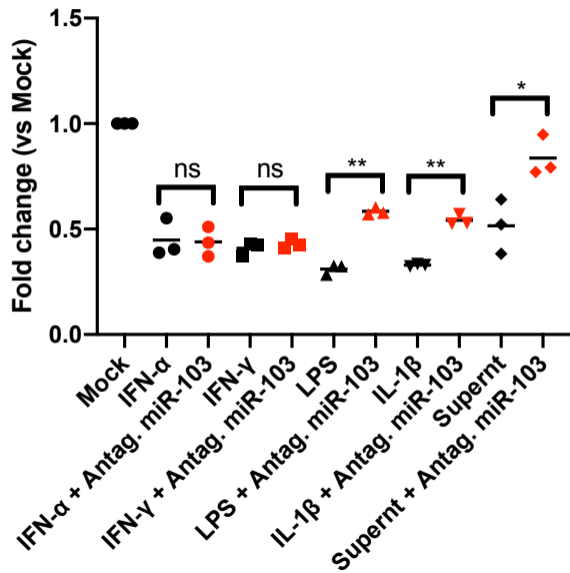

cd4

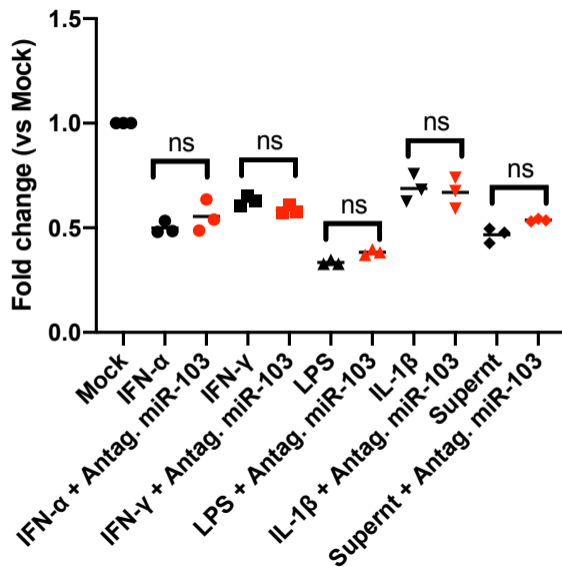

Supplement: FIG S1 [file mBio.02314-20-sf001.pdf]

## MiR-103

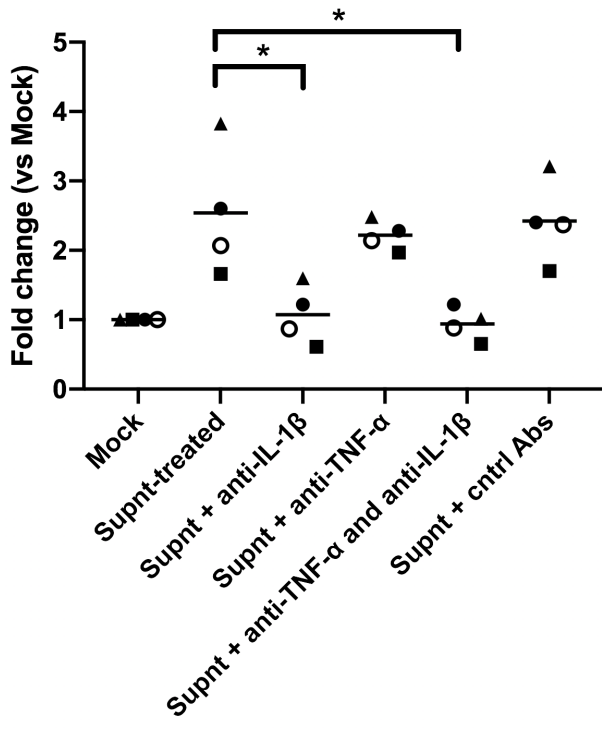

## MiR-222

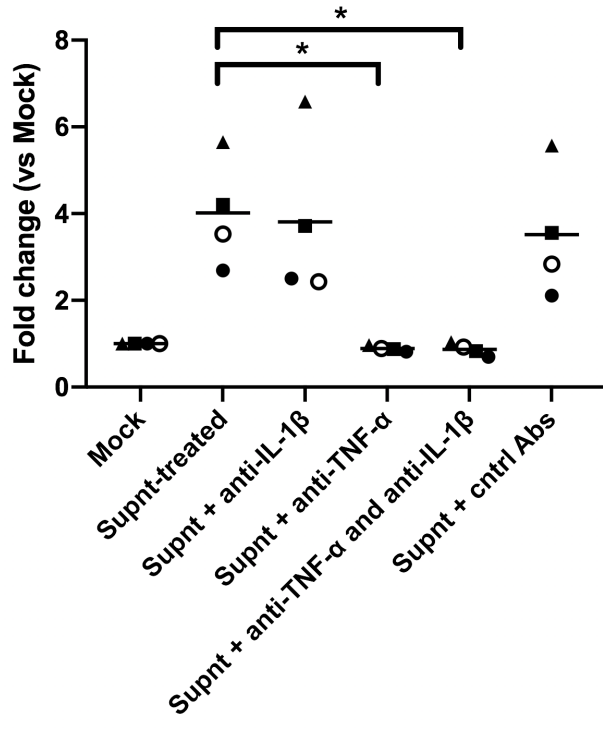

Supplement: FIG S2 [file mBio.02314-20-sf002.pdf]
