# Supplementary material for: Interleukin-1β Triggers p53-Mediated Downmodulation of CCR5 and HIV-1 Entry in Macrophages through MicroRNAs 103 and 107
Source: mBio. 2020 Sep 29;11(5):e02314-20. doi: 10.1128/mBio.02314-20 (PMC7527731; doi:10.1128/mBio.02314-20)
Supplement: TABLE S2 [file mBio.02314-20-st002.pdf]

Table S2 - Oligonucleotides used in this study.

NAME	OLIGONUCLEOTIDE	PURPOSE
5'CCR5-UTR	TACAAGAGCTCTTGTGACACGGACTCAAGTGG	LucCCR5-3'UTR
3'CCR5-UTR	TACAAACGCGTTTATTTTAGAAGGTGTTTTTATTAGATG	LucCCR5-3'UTR
CCR5mutFwd	CTGAAAAACTAAGCTTACAGCTGCCCAGTG	Seed mutations
CCR5mutRev	CACTGGGCAGCTGTAAGCTTAGTTTTTCAG	Seed mutations
miR-222-3p-L	CAGATGTATCAAGCTCTCCAGGTACAGTTGGTACCTGACTCCACGCACCCAG	qPCR
miR-222-3p-5'	CAGATGTATCAAGCTCTCCAGGTACAGT	qPCR
miR-222-3p-3’	GGAGCTACATCTGGCTACTGGGT	RT loop
miR-103-3p-5'	ACAATGTCAAGCTCTCCAGGTACAGT	qPCR
miR-103-3p-3'	GGAGCAGCATTGTACAGGGCT	qPCR
miR-103-3p-L	TACAATGTCAAGCTCTCCAGGTACAGTTGGTACCTGACTCCACGCTCATAG	RT loop
miR-107-5’	ACAATGGCTAGCTATGCAGGTACAG	qPCR
miR-107-3’	GGAGCAGCATTGTACAGGGCT	qPCR
miR-107-L	TACAATGGCTAGCTATGCAGGTACAGTTGGTACCTGACTCTTGTTTGATAG	RT loop
gapdh-5'	GCCATCAATGACCCCTTCAT	qPCR
gapdh-3'	TTGACGGTGCCATGGAATTT	qPCR
cd4-F	GTCCCTTTTAGGCACTTGCTTCT	qPCR
cd4-R	TCTTTCCCTGAGTGGCTGCT	qPCR
ccr5-F	CAAAAAGAAGGTCTTCATTACACC	qPCR
ccr5-R	CCTGTGCCTCTTCTTCTCATTTCG	qPCR
p53-F	GCCCAACAACACCAGCTCCT	qPCR
p53-R	CCTGGGCATCCTTGAGTTCC	qPCR
p21-F (A)	TCCTCATCCCGTGTTCTCCT	qPCR
p21-R (A)	ACAAGTGGGGAGGAGGAAGT	qPCR
miR-103C FWD	AATTCAGCAGCATTGTACAGGGCTATGAAAAAAG	pMIR103
miR-103C REV	GATCCTTTTTTCATAGCCCTGTACAATGCTGCTG	pMIR103
miR-107C FWD	AATTCAGCAGCATTGTACAGGGCTATCAAAAAAG	pMIR107
miR-107C REV	GATCCTTTTTTGATAGCCCTGTACAATGCTGCTG	pMIR107
miR-103/107-5'	CGCAGAGCAGCATTGTACAG	qPCR (absolute)
miR-103-3p-3'	GGTCCAGTTTTTTTTTTTTTTTCATAG	qPCR (absolute)
miR-107-3’	GGTCCAGTTTTTTTTTTTTTTTGATAG	qPCR (absolute)
miR-103 mimic	AGCAGCATTGTACAGGGCTATGA	
miR-107 mimic	AGCAGCATTGTACAGGGCTATCA	
miR-103 antag.	GCCCTGTACAATGCTGCT	
miR-107 antag.	GATAGCCCTGTACAATG	
